# Health Service delivery and training at Makerere University College of Health Sciences: The role of the academic health centers' network

**DOI:** 10.4314/ahs.v22i2.10S

**Published:** 2022-08

**Authors:** Damalie Nakanjako, Josaphat Byamugisha

**Affiliations:** 1 Department of Medicine, School of Medicine, Makerere University College of Health Sciences, Kampala, Uganda; 2 Department of Obstetrics and Gynecology, School of Medicine, Makerere University College of Health Sciences, Kampala, Uganda; 3 Makerere University Health Services, Makerere University College of Health Sciences, Kampala, Uganda

**Keywords:** Academic Health Centers, Clinical training, Health care delivery, Health professions education

## Abstract

Academic Health Centers provide a network of clinical training facilities for high quality service delivery, within clinical training and research settings. At an academic health/medical center, education, research, and clinical care are combined to provide the best possible clinical care, which uses cutting-edge technologies, resources and therapies other community hospitals may not have available.

This paper summarizes the network of teaching hospitals for health care training in the last century of Makerere University College of Health Sciences, its local and regional impact, lessons learned and future of actively engaging the network of academic health centers to expand clinical training and research opportunities to meet the needs of the 21^st^ Century populations.

Medical schools and training hospitals need to integrate of their roles in research, education, and patient care to provide evidence-based interventions and innovations to both treat illness and improve health.

## Background

Academic Health Centers may be best described as complexes of medical schools and other health professional schools (nursing, Pharmacy. Dentistry, and allied health) affiliated with each other and with teaching hospitals and other research and clinical facilities^1^. Academic Health Centers provide a network of clinical training facilities for high quality service delivery, within clinical training and research settings. At an academic health/medical center, education, research, and clinical care are combined to provide the best possible clinical care, that uses cutting-edge technologies, resources and therapies other community hospitals may not have available^2^. This paper summarizes the successes in health care training in the last century through a network of teaching hospitals at Makerere University College of Health Sciences (MakCHS). We also highlight lessons learned and the preparedness to sustain improvements in health professions training, innovation and service delivery for the 21^st^ century communities.

MakCHS is a member of the Association of Academic-Health Centers International (AAHCI) Eastern Africa Regional Office (AAHCI-EA) which is an integral part of the Association of Academic Health Centers (AAHC) that was founded in 1969 to advance health and well-being through the dynamic leadership of academic health centers in the United States to provide opportunities for shared knowledge, capacity-building, and collaborative initiatives and efforts across regional academic health centers^3^. AAHCI aims to bring together institutions around the world that serve the academic health center mission and share a global vision of enhancing health and well-being worldwide^4^.

Since its inception in 1922, Makerere University medical school has trained thousands of doctors at Mulago hospital and the clinical setting has birthed many innovations in clinical care and research including the first description of Burkitt Lymphoma by Dennis Burkitt in 1958 as a sarcoma of the jaw^5^–^8^, which led to the establishment of a dedicated cancer research institute, the Uganda Cancer Institute in 1967 9. Subsequently Kaposi's sarcoma was described in Uganda in 1970 by Kyalwazi SK in early immunological studies^10^. In 1985, “Slim disease” was described by Ugandan scientists as a new disease associated with HTLV-III infection^11^. Similarly epidemic Kaposi Sarcoma was described among children who presented to the Uganda Cancer Institute between 2004–2007 12. In response to the HIV epidemic that was associated with high risk of transmission from HIV-infected pregnant mothers to their unborn babies, a landmark HIVNET 012 study was conducted in Uganda in 1999. HIVNET 012 showed that single-dose Nevirapine at onset of labor and a single dose to the infant led to a 42% reduction in maternal to child HIV transmission, providing the developing world with a cheap and simple option to protect thousands of children born by HIV-infected mothers^13^, ^14^. Furthermore, clinical trials were conducted at MakCHS' hub for PMTCT care and research, the Makerere University John Hopkin's collaboration^14^, ^15^. Makerere University scientists continued to contribute to the national and regional response to the HIV/AIDS epidemic through innovative approaches to treatment of HIV/AIDS and co-infections including tuberculosis, and cryptococcal meningitis^16^–^20^. MakCHS scientists continue to provide technical expertise to the national health priorities including contributions to the national response to the COVID-19 pandemic^21^, ^22^.

Clinical care training and service delivery was traditionally offered at Mulago national referral and teaching hospital, which hosted the Uganda Heart Institute and the Uganda Cancer Institute. With the various advances in clinical care and growth of the Ugandan population served by these facilities the latter have developed into centers of excellence. Clinical care and training were further expanded by the establishment of Kawempe National Referral Hospital and specialized women and neonatal hospital for women, Kiruddu National Referral hospital for internal medicine, and Butabika Hospital for mental health care. In addition, Makerere University has expanded the University Health Care services to include the University Hospital (formerly named sickbay) which currently hosts the most specialized optometry unit in the country, and a dental school; in addition to other specialized services offered by faculty in their respective disciplines. Makerere University has embarked on building capacity of the University hospital Intensive Care services that were much needed during the COVID-19 pandemic.

Furthermore, through the community-based education, research and service (COBERS) program for health science students, MakCHS accredited over 60 health care facilities in all parts of the country to promote community-based learning, leadership and interprofessional education as a strategy to produce medical graduates with the skills, passion and relevance needed to serve the respective communities in Uganda. All these clinical care facilities constitute the clinical training network to expand clinical care and training. Clinical training settings provide fertile ground for experts to pass on hands-on skills to medical trainees under their supervision as well as opportunities for innovations in clinical care approaches under a controlled environment. The centers of excellence also provide opportunities for clinical specialization, to meet the increasing demand of patients who require specialized care; including interventional cardiology, gastroen-terology, oncology, hematology, neurology, neurosurgery and neonatology, among others.

The clinical care, training and research platforms at MakCHS present opportunities for vibrant clinical and academic learning for students and faculty, that should be harnessed through the regional network of Academic Centers. Regular interactions through student and faculty mobility programs, clinical grand rounds, case conferences, tumor boards, among others, should be optimized to promote continuous mutual learning between teaching hospitals within regional and international academic health centers networks.

## Impact of MakCHS training and health care platform

- Academic health centers are leading contributors to national and regional responses to emerging global health challenges. During the COVID-19 pandemic several clinical centers served by Makerere University faculty contributed to the COVID-19 response and many teaching units and faculty were re-purposed to support the COVID-19 response. The Infectious Diseases faculty and pulmonologists supported case management and clinical trials to generate relevant evidence, Intensive care and High dependency units (Anesthesiologists & Critical care Physicians) supported critically ill patients. The Psychiatrists and psychologists were overwhelmed with mental health complications of the diseases while community engagement through the Public Health initiatives helped to increase community awareness and protection through vaccination and standard operating procedures to prevent transmission.
- Laboratory strengthening programs at the University in collaboration with the Ministry of Health laboratories increased access to COVID-19 tests in the country to furnish the national task force with numbers and trends of the disease, to inform appropriate guidelines and policies. Similarly academic institutions contributed a lot to training of health workers in the use of standard operating procedures (SOP), as well as several virtual training modules that were developed and implemented; for example, management of COVID-19 in pregnancy.
- The role of mental health clinics at Mulago and Butabika hospitals cannot be underestimated given the fact that local and global pandemics including HIV/AIDS, COVID-19 and the emerging risk of non-communicable diseases pose a major threat to the mental and psychological wellbeing of the community. Communities are at a high risk of psychological and mental health problems including depression, anxiety, suicide, and post-traumatic stress disorder due to the disease, loss of employment, fear of acquiring the disease, separation from families, lack of proven treatment, fear of death, and loss of loved ones. Similarly, Health care providers are at a high risk of psychological disturbances and mental health problems due to stress related to the disease, death of patients, lack of adequate personal protective equipment, lack of proven treatments, long shifts without sleep, separation from family, and fear of death.
- Professional, strategic and dynamic leadership is critical to prepare academic health centers in Uganda and sub-Saharan Africa region to embrace the changes necessary to create an environment with cohesive, innovative, multi-disciplinary approaches to training and health care delivery. With the recent accreditation of MakCHS as a Regional Foundation for advance of International Medical Education and Research (FAIMER) Institute in East Africa, MakCHS will consolidate and expand its efforts to promote high quality health professions education through programmatic and research activities to enhance educational resources to teach health professionals and improve the health of communities served by the faculty, graduates and all stakeholders.

## Lessons Leaned

- Information and telecommunications technology is a major force in cultivating a more informed consumer and can engage patients in exerting more direction and control over their care, altering their interactions with and expectations from clinicians. Expanding technology in the health care system will complement the good clinical skills and instruction to optimize the quality and safety of clinical services.
- Health care, like all industries, is affected by globalization that speeds the transfer of knowledge, but also the transmission of disease. Teaching hospitals need to be equipped for innovation, research and training tools, to develop the agility required to address the ever-emerging challenges to global health.
- With physicians, nurses, pharmacists, researchers, and teachers all working in unison, patients have better access to the latest medical breakthroughs and clinical trials that are not available at other hospitals^2^.
- Inter University Collaboration between Universities in high income countries with those in Low-income countries enables easy transfer of skills to the medical faculty. The regional and international network of academic health centers provides a wider community of learning from sharing clinical evidence.
- Faculty in academic centers have more tendencies to professional competence growth since academic growth involves teaching, service delivery research and publication. For example, a surgeon who starts as an assistant lecturer undertaking stem cell research may find themselves performing organ transplantation by the time they become senior lecturer because of the research and skills development program in which they participated.
- Funding agencies should work together to promote multi-disciplinary collaborations with a mix of scientists doing different types of research to answer the important questions of science and health; collectively linked through the academic centers.
- There is a need to be intentional about preparedness for the next century. A dedicated fund should be created to foster innovations in the infrastructure, methods and approaches used to prepare health professionals for the 21^st^ Century and beyond.
- The future presents new demands on medical education and health care including keeping up with the fast advances in biomedical and technological approaches to health care delivery. We need to equip the infrastructure to utilize the expertise of our trained specialists, to enable them to train other upcoming specialists through graduate training and specialized clinical fellowship programs.

## Future plans

- Expand clinical specialty training: In the last decade, several Ugandan specialists have received super-specialized training at academic centers abroad in collaboration with MakCHS, in an effort to build the critical mass of super-specialized clinicians to train others who will sustain the specialized hospitals being developed. MakCHS already has specialists in interventional cardiology diagnostic cardiology hematology, gastroenterology, pharmacokinetics, immunology, molecular biology, neurosurgery, neonatology, gynoncology, among others; and subsequently specialized clinical fellowship programs are being accredited to promote local training. Similarly, the Medical Doctorate program at MakCHS has received increasing applicants following the increasing facilities and trainers in specialized clinical areas that were previously accessed abroad. Many graduates of clinical specialty programs are increasingly finding it more feasible to return home and practice their skills if the infrastructure and environment are conducive^23^.
- Train in additional relevant clinical disciplines: Makerere University plans to expand health services, training and research to include Nano-medicine, Military medicine, Transplant medicine, Marine medicine, Robotic surgeries, Stem cell transplants, Sports medicine, Aviation medicine, Genetics and Precision medicine; to meet the increasing demands of these services in Uganda and East Africa region.
- Upgrade infrastructure for clinical training, care and research: To expand the infrastructure to support these much-needed services, Makerere University has embarked on a building program to expand the health care capacity of the hospital. This was in part prompted by the big unmet need to expand teaching facilities to keep up with the new advances in medical care, instrumentation and digitalization required for the 21^st^ Century. The response COVID-19 pandemic exposed several gaps in specialized care that the MakCHS academic centers will collectively address to improve preparedness for emerging and re-emerging health care needs; including high dependency units, intensive care and other services that Ugandans could no-longer access from abroad due to travel COVID-19 related restrictions especially organ transplants and complex neurosurgical procedures. With new training programs in immunology and molecular biology, and the tissue biobank MakCHS is preparing to offer organ transplant services and related translational research.

## Conclusion

Medical schools and training hospitals need to integrate their roles in research, education, and patient care to provide evidence-based interventions and innovations to both treat illness and improve health. Resources should be dedicated to foster innovation in the infrastructure, methods and approaches used to prepare health professionals for the 21^st^ Century and beyond. Advances in technology should be harnessed to improve apprenticeship and specialized health care and research. The network of academic centers provides a wider platform for mutual learning from evidence -based clinical care.

## References

[1] LT K, Institute of Medicine (US) Committee on the Roles of Academic Health Centers in the 21^st^ Century (2004). Academic Health Centers: Leading Change in the 21st Century.

[2] Walusansa V, Okuku F, Orem J (2012). Burkitt lymphoma in Uganda, the legacy of Denis Burkitt and an update on the disease status. Br J Haematol.

[3] Association of Academic Health Ccenter-East Africa Region (2019). https://www.aahcdc.org/AAHCI-Regional-Offices/AAHCI-Eastern-Africa-Regional-Office%20newsletter.

[4] (AAHCI) AoAHaI (2021). https://www.aahcdc.org/AAHCI-Regional-Offices/AAH-CI-Eastern-Africa-Regional-Office.

[5] Walusansa V, Okuku F, Orem J (2012). Burkitt lymphoma in Uganda, the legacy of Denis Burkitt and an update on the diseases status. British Journal of Haematology.

[6] Burkitt D (1958). A sarcoma involving the jaws in African children. Br J Surg.

[7] Burkitt D (1962). A “tumour safari” in East and Central Africa. Br J Cancer.

[8] Burkitt D (1962). A tumour syndrome affecting children in tropical Africa. Postgrad Med J.

[9] Walusansa V, Okuku F, Orem J (2012). Burkitt lymphoma in UGANDA, the legacy of Denis Burkitt and an update on the disease status. British Journal of Haematology.

[10] Master SP, Taylor JF, Kyalwazi SK, Ziegler JL (1970). Immunological studies in Kaposi's sarcoma in Uganda. Br Med J.

[11] Serwadda D, Mugerwa RD, Sewankambo NK, Lwegaba A, Carswell JW, Kirya GB (1985). Slim disease: a new disease in Uganda and its association with HTLV-III infection. Lancet.

[12] Gantt S, A K, Wald A, Walusansa V, Corey L, Corey C (2010). Clinical presentation and outcome of epidemic Kaposi Sarcoma in Ugandan children. Pediatric blood & cancer.

[13] Guay LA, Musoke P, Fleming T, Bagenda D, Allen M, Nakabiito C (1999). Intrapartum and neonatal single-dose nevirapine compared with zidovudine for prevention of mother-to-child transmission of HIV-1 in Kampala, Uganda: HIVNET 012 randomised trial. Lancet.

[14] Musoke PM, FA M (2000). Prevention of HIV Mother to Child Transmission: A review. AIDS Reviews.

[15] Petra Study T (2002). Efficacy of three short-course regimens of zidovudine and lamivudine in preventing early and late transmission of HIV-1 from mother to child in Tanzania, South Africa, and Uganda (Petra study): a randomised, double-blind, placebo-controlled trial. Lancet.

[16] Boulware DR, Meya DB, Muzoora C, Rolfes MA, Huppler Hullsiek K, Musubire A (2014). Timing of antiretroviral therapy after diagnosis of cryptococcal meningitis. N Engl J Med.

[17] Kwarisiima D, Kamya MR, Owaraganise A, Mwangwa F, Byonanebye DM, Ayieko J (2017). High rates of viral suppression in adults and children with high CD4+ counts using a streamlined ART delivery model in the SEARCH trial in rural Uganda and Kenya. J Int AIDS Soc.

[18] Nakanjako D, Mayanja-Kizza H, Ouma J, Wanyenze R, Mwesigire D, Namale A (2010). Tuberculosis and human immunodeficiency virus co-infections and their predictors at a hospital-based HIV/AIDS clinic in Uganda. Int J Tuberc Lung Dis.

[19] Nakanjako D, Ssinabulya I, Nabatanzi R, Bayigga L, Kiragga A, Joloba M (2015). Atorvastatin reduces T-cell activation and exhaustion among HIV-infected cART-treated suboptimal immune responders in Uganda: a randomised crossover placebo-controlled trial. Trop Med Int Health.

[20] Nakiyingi L, Ssengooba W, Nakanjako D, Armstrong D, Holshouser M, Kirenga BJ (2015). Predictors and outcomes of mycobacteremia among HIV-infected smear-negative presumptive tuberculosis patients in Uganda. BMC Infect Dis.

[21] Kiragga AN, Ktayimbwa J, Galiwango R, AK M (2020). POLICY BRIEF: Experts issue critical advice on lockdown lifting.

[22] PAN AFRICA (2020). Half of Uganda's Covid-19 task force are Makerere staff.

[23] Kendall DP (2012). Medical Brain Drain in Uganda: Causes and Potential Remedies. Annals of Global Health.

